# Editorial: Positive Psychology and Learning a Second or Third Language

**DOI:** 10.3389/fpsyg.2020.599326

**Published:** 2020-10-23

**Authors:** Amado M. Padilla, Xinjie Chen, J. Lake

**Affiliations:** ^1^Graduate School of Education, Stanford University, Stanford, CA, United States; ^2^Department of International Liberal Arts, Fukuoka Women's University, Fukuoka, Japan

**Keywords:** positive psychology, second language learning and acquisition, third language learning and acquisition, bilingualism and multilingualism, bicultural bilinguals

Positive Psychology (PosPsy) has slowly gained traction in the study and practice of psychology since it was first introduced by (Seligman and Csikszentmihalyi, 2000). Unlike traditional psychology, which focuses on problematic behaviors, PosPsy examines topics such as happiness, well-being, flourishing, and resilience. Interest in PosPsy has moved into other fields of study including second language acquisition (SLA) research. In the past SLA researchers have spent considerable time studying impediments such as stress and anxiety to explain difficulties in learning a second language. Today a quickly growing trend among SLA researchers' is to see the value of PosPsy constructs. With this increased recognition has come a reorientation among SLA researchers in how best to re-imagine second or even a third language acquisition. Given the flattening of our world brought about by the Internet, bi/multilingualism has become commonplace and even a necessity for students in many countries around the world.

Many language researchers who see the value of studying second/foreign language acquisition from a more open, appreciative and positive perspective welcome this exciting new orientation in SLA research. Researchers and educators with a PosPsy orientation are more prone to explore how learning a new language can bring joy, interest, and excitement to learners because of the new worlds it opens up for the burgeoning bi/multilingual. This is not to say that there are not difficulties associated with language study. However, when a learner overcomes personal challenges and begins to use a new language there are moments of exhilaration and accomplishment.

Earlier research in SLA largely discussed the negative elements in SLA, such as learner anxiety. Today a growing number of researchers have recognized the important impact of promoting learner positive traits and strengths in SLA. For example, researchers have highlighted the importance of alternative methods to facilitate positive language learning experiences that ideally minimize second language learners' anxiety and optimize language acquisition. Instead of focusing on negative emotions (e.g., anxiety, stress, failure) during language learning, PosPsy seeks to develop positive emotions, greater engagement, and meaningful language learning experiences for second language learners.

SLA researchers who employ a PosPsy orientation are beginning to make significant contributions to both SLA theory and application to facilitate second and third language learning. Theoretically, applying PosPsy constructs and methods to SLA help language researchers better understand the field of SLA through a new and positive lens. Some of this work offers guidelines for ways to think about the role of motivation in learning a new language. From a pedagogical perspective, PosPsy SLA research offers language educators insights and strategies for improving learners' positive emotional preparation for learning. With meaningful teaching and learning interactions the potential for improved second language proficiency is present. Notably, PosPsy studies of SLA are providing evidence-based strategies for teachers regarding the creation of positive L2 learning environments that heighten learners' motivation, perseverance, resilience, and positive emotions for a long-term meaningful language learning experience.

The overarching goal for organizing this eBook was to offer groundbreaking work in second and third language research from an international group of researchers who use a PosPsy orientation to guide their work. This eBook includes three exciting directions in PosPsy SLA research. First, theoretical reviews that range from young children to secondary school and university students who require a second language to advance academically, as well as mature adults offered the opportunity to learn a second language for the purpose of enjoyment, but also memory improvement. Second, empirically grounded studies that link PosPsy with SLA. These studies offer objective evidence-based research on ways that constructs taken from PosPsy (e.g., mindsets, self-efficacy) can be used in controlled studies to examine learning a second or third language. Third, practical classroom oriented studies that offer data on how to enable learners to channel negative emotions (e.g., anxiety) about learning and performing in a second/third language so they can experience positive flow in learning.

A secondary goal was to advance the literature by stressing the interdisciplinary link between PosPsy and SLA through presenting the work of researchers representing a wide range of cultural and linguistic contexts. We hope this collection encourages future efforts to bridge the gap between second and third language learning/teaching and PosPsy by providing an interdisciplinary and international forum for the science and application of SLA research.

## Author Contributions

AP, XC, and JL contributed equally to this special topic. All authors contributed to the article and approved the submitted version.

## Conflict of Interest

The authors declare that the research was conducted in the absence of any commercial or financial relationships that could be construed as a potential conflict of interest.
